# Pyogenic (Suppurative) Flexor Tenosynovitis: Assessment and Management

**Published:** 2016-02-12

**Authors:** Renee L. Barry, Nicholas S. Adams, Matthew D. Martin

**Affiliations:** ^a^Michigan State University College of Human Medicine, Grand Rapids, Mich; ^b^Grand Rapids Medical Education Partners Plastic and Reconstructive Surgery Residency, Grand Rapids, Mich; ^c^Hand Surgery Centre, Grand Rapids, Mich

**Keywords:** pyogenic flexor tenosynovitis, Kanavel signs, hand infection, penetrating trauma, tendon sheath

## DESCRIPTION

A 62-year-old diabetic male patient presented to the emergency department with right index finger pain and swelling 4 days after sustaining a puncture wound. On physical examination, the digit was held in a flexed position, and there was pain with passive extension, fusiform swelling, and tenderness over the flexor tendon.

## QUESTIONS

1. **What are the causes and most common pathogens associated with suppurative flexor tenosynovitis?**
2. **What are Kanavel signs?**
3. **How should suppurative flexor tenosynovitis treated?**
4. **What are the potential complications of suppurative flexor tenosynovitis?**

## DISCUSSION

Pyogenic (suppurative) flexor tenosynovitis (PFT) is a serious diagnosis accounting for up to 10% of acute hand infections.^1^ This purulent infection is the result of bacterial invasion of the flexor tendon sheath, a closed anatomic space between the visceral epitenon layer and the outer parietal layer. Accordingly, pressure buildup leads to distention between the visceral and parietal layers and subsequent disruption of the neighboring anatomical barriers. The fascial spaces of the hand and adjacent bursae can then harbor infection; communication with the forearm may be established through the space of Parona.^1^^-^5 PFT most frequently corresponds with a recent history of penetrating trauma, but it has also been documented in cases of hematogenous seeding.^2^^,^^4^^,^^5^ Common isolates include *Staphylococcus aureus* (most frequently identified), methicillin-resistant *Staphylococcus aureus*, *Staphylococcus epidermidis*, group A *Streptococcus*, and *Pseudomonas aeruginosa*. If a history of human or animal bite is described, *Eikenella corrodens* or *Pasteurella multocida* should be considered as the source of infection.^2^

**Figure 1:**
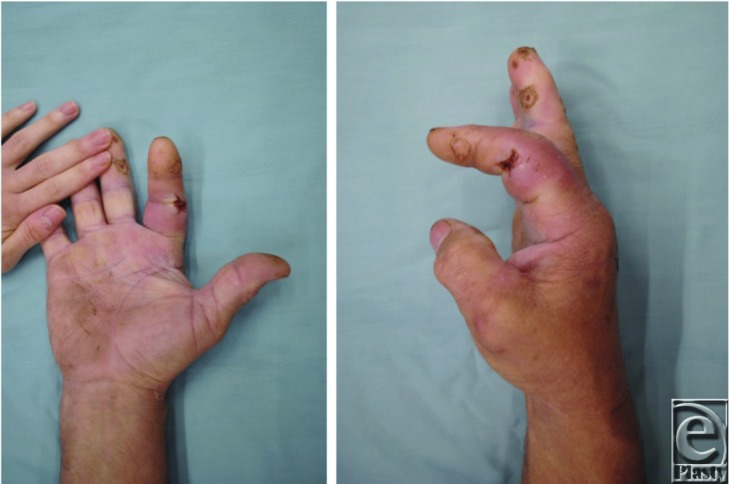
Anteroposterior (a) and lateral (b) photographs demonstrating fusiform swelling and flexed posture of the right index finger.

**Figure 2:**
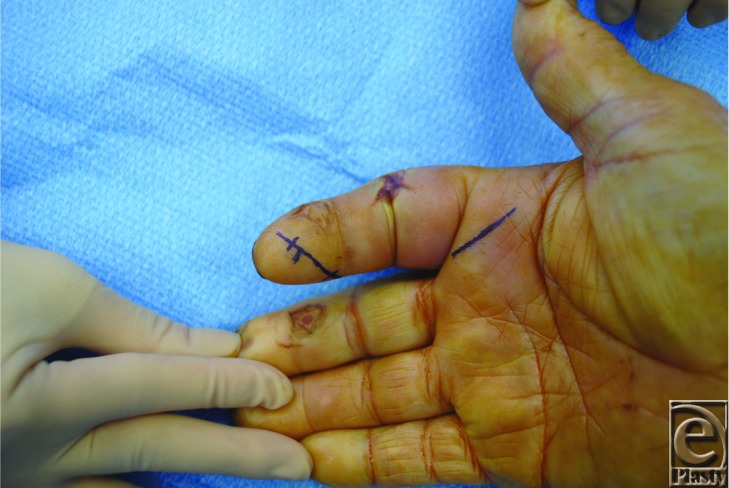
Surgical markings for closed tendon sheath irrigation overlying the A1 pulley and distal phalanx to allow for proximal and distal access of the flexor sheath. The sheath can then be flushed using a 16-gauge angiocatheter or a 5-French pediatric feeding catheter.

PFT is primarily a clinical diagnosis. Although white blood cell count, erythrocyte sedimentation rate, and C-reactive protein level can aid in diagnosis, these tests are considered sensitive with a low negative predictive value and specificity.^6^ Kanavel^3^ described 4 cardinal signs that are used to differentiate PFT from other infections. These signs include (1) fusiform swelling of the finger (Fig 1a), (2) exquisite tenderness over the course of the sheath, (3) exquisite pain on passive extension, and (4) flexed posture of the digit (Fig 1b). The specificity and sensitivity of these hallmarks have not been established in the literature; however, they remain a useful clinical tool for diagnosis.^3^

If PFT is detected within 48 hours, nonsurgical management with intravenous antibiotics and elevation of the hand may be appropriate. The affected hand should be closely monitored. If no improvement or worsening symptoms are detected after 24 hours, surgical intervention is necessary.^2^^,^^4^ Either closed tendon sheath irrigation (Fig 2) or open irrigation and debridement technique may be used, with the later deemed preferential for severe cases. In the closed tendon sheath technique originally described by Neviaser,^7^ a proximal zigzag incision is made over the metacarpal neck, which allows for exposure of the tendon sheath. An angiocatheter is threaded into the flexor tendon sheath proximal to the A1 pulley and the sheath is irrigated with sterile normal saline. Alternatively, a 5-French pediatric feeding catheter may be used for irrigating the tendon sheath. A distal incision is made to allow placement of a Penrose drain to allow for distal drainage of irrigation solution.^7^ With an open irrigation and debridement, Brunner-type incisions are used to expose the tendon sheath and allow for irrigation and drainage.^2^^,^^4^^,^^5^

Prompt treatment of PFT does not eliminate the potential for complications. Previous studies report a 10% to 25% incidence of residual digital stiffness resulting from flexor tendon adhesions, joint capsular thickening, breakdown of the pulley system/flexor sheath, or surgical impediments.^2^ Further complications may include spread of infection, necrosis of tendon and tendon rupture, osteomyelitis, and amputation. Factors increasing the likelihood of amputation include delayed treatment, digital ischemia at presentation, subcutaneous purulence, age greater than 43 years, and comorbidities including diabetes mellitus, renal failure, and peripheral vascular disease.^8^

PFT requires timely recognition and intervention by the clinician. Health care providers should be familiar with the 4 hallmark signs depicted by Kanavel. Failure to appropriately administer intravenous antibiotics and manage operatively can culminate in complications including spread of infection and digit amputation.

## Figures and Tables

**Figure .**
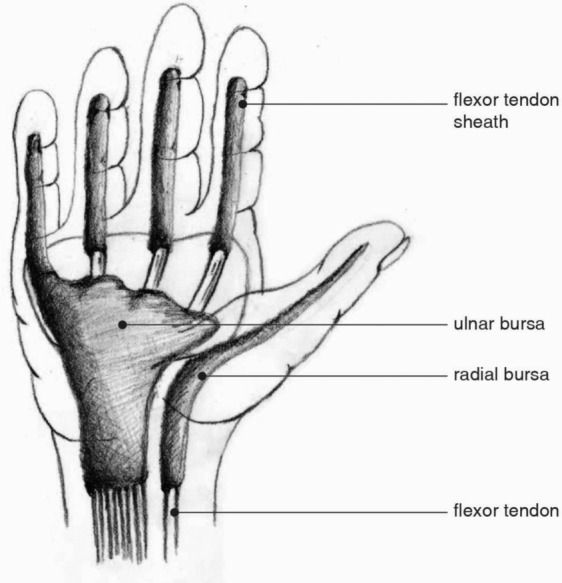
Illustration of the flexor tendon sheaths and bursae of the hand. The interconnections between the bursae and tendon sheaths allow for spread of infections to adjacent deep spaces of the hand.
